# Use of a home-use test to diagnose HIV infection in a sex partner: a case report

**DOI:** 10.1186/1756-0500-5-440

**Published:** 2012-08-15

**Authors:** David A Katz, Matthew R Golden, Joanne D Stekler

**Affiliations:** 1 Department of Epidemiology, University of Washington, Seattle, WA, USA; 2 Departments of Epidemiology and Medicine, University of Washington, Seattle, WA, USA; 3 HIV/STD Program, Public Health Seattle & King County, Seattle, WA, USA

**Keywords:** HIV screening, Home-use tests, Men who have sex with men, Serosorting, Rapid HIV testing

## Abstract

**Background:**

Home-use HIV tests have the potential to increase testing and may be used by sex partners to inform sexual decision-making. To our knowledge, this is the first report of an individual diagnosed with HIV using a home-use test with a sex partner.

**Case presentation:**

We are conducting a randomized controlled trial of home self-testing for HIV using the OraQuick ADVANCE^®^ HIV-1/2 Antibody Test on oral fluids. In 2011, a 27-year-old, homeless, Latino man who has sex with men not enrolled in the trial (the case) reported receiving a reactive result from a diverted study kit. When interviewed by study staff, the case reported that, 11 months prior, he had unprotected anal sex with a trial subject without discussing HIV status. Afterwards, the subject asked the case if he would like to test, performed the test, and disclosed the reactive result. The case reported altering his behavior to decrease the risk of HIV transmission to subsequent partners and sought care two months later.

**Conclusions:**

This case demonstrates that home-use HIV tests will be used by sex partners to learn and disclose HIV status and inform sexual decision-making. It also highlights concerns regarding the absence of counseling and the potential for delayed entry into HIV care. Additional research must be done to determine under what circumstances home-use tests can be used to increase awareness of HIV status, how they impact linkage to care among persons newly diagnosed with HIV, and whether they can be safely used to increase the accuracy of serosorting.

## Background

HIV testing is not only the entry point into care for HIV-infected individuals but also an effective prevention intervention because most newly-diagnosed individuals will alter their behavior to decrease transmission to others [1] and antiretroviral therapy can reduce infectiousness [2].

In 1996, the U.S. Food and Drug Administration (FDA) approved the Home Access HIV-1 Test System (Home Access Health Corporation; Hoffman Estates, IL), a home collection kit for HIV antibody testing. With home collection, testers send self-collected dried blood spots to a laboratory and learn their test results by phone. In the first year it was available, 174,316 kits were sold and 0.9% persons were positive, 49% of whom had never tested before [3]. Few have used these kits, however, in part due to concerns about accuracy and cost [3-5].

Home-use tests, through which testers use a rapid HIV test on self-collected oral fluids or blood to learn their HIV status at home, have been posited as an alternative method to increase HIV testing [6-8] and thereby decrease the time HIV-infected individuals are unaware of their status and potential for transmission. However, there are concerns about the potential for misinterpretation of test results, greater number of false-negative test results during the relatively long window period of currently available rapid tests, reduced access to risk-reduction counseling, decreased screening for sexually transmitted infections (STIs), missed opportunities to link HIV-infected persons into care or to perform partner services, and reduced accuracy of HIV surveillance [7-10]. When home-use tests are available over-the-counter, they may also be used by sex partners to inform sexual decision-making [11].

In July 2012, the FDA approved the first over-the-counter home-use HIV test, the OraQuick^®^ In-Home HIV Test (OraSure Technologies, Inc., Bethlehem, PA). In premarketing studies of untrained users in uncontrolled settings, the test was 93.0% sensitive, 99.98% specific, and had a window period of 3 months [7]. No industry-sponsored studies were planned to address whether at-risk individuals will be more likely to test or to test more often if home-use tests are available; concerns regarding reductions in counseling, linkage to care, and accuracy of surveillance; or home testing by sex partners [7].

The iTest Study is an ongoing, NIH-funded, randomized controlled trial to evaluate the impact of access to home-use tests on HIV testing frequency. HIV-negative men who have sex with men (MSM) are randomized to home self-testing using the OraQuick ADVANCE^®^ Rapid HIV-1/2 Antibody Test (which uses the same technology as the approved home-use test) on oral fluids or to standard HIV testing for 15 months (Clinicaltrials.gov Identifier NCT01161446). Participants randomized to home self-testing are trained to use the test at enrollment and receive one test kit at a time for use on themselves only. Study test kits include the test and study-specific instructions, counseling materials, and local resources and are provided at no cost to participants.

We report the diversion of a study test kit to test a trial subject’s sex partner, whose rapid HIV test on oral fluids was reactive. To our knowledge, this is the first report of an individual diagnosed with HIV using a home-use test with a sex partner.

## Case presentation

In 2011, a 27-year-old, homeless, gay-identified Latino MSM not enrolled in the trial (the case) was seen at the Public Heath – Seattle & King County (PHSKC) STD Clinic and reported receiving a reactive result from a diverted study kit. This case study received ethical approval from the University of Washington Institutional Review Board, and written informed consent was obtained from the case for a semi-structured interview about his experience testing with a sex partner and subsequent entry into care.

The case reported that, 11 months prior to this interview, he had unprotected anal sex and used methamphetamine with a trial subject without discussing HIV status. Afterwards, the trial subject asked the case if he wanted to use a study test kit. The case last tested 13 months earlier. The trial subject collected the case’s oral fluid sample, performed the test, and disclosed the reactive result. The case thought it was an acceptable way to learn his HIV status. The case and trial subject did not engage in further sexual activity. Neither confirmatory testing nor post-exposure prophylaxis was discussed.

The case did not seek confirmatory testing immediately. He assumed the results were definitive and did not want antiretroviral therapy. He reported being subsequently more likely to disclose his HIV status and use condoms with sex partners. Two months later, he sought confirmatory testing at the PHSKC STD Clinic. He initiated HIV care two months after confirmatory testing. Six months after the home test, his CD4+ T-cell count was 396 cells/mL, and his HIV RNA level was 15,080 copies/mL. One year after the home test, he was engaged in HIV care [12] but not receiving antiretroviral therapy.

This case highlights potential benefits and risks of home-use HIV tests. First, home-use tests have the potential to increase testing due to convenience, anonymity, and reduced time needed to test [6-8]. Furthermore, the test result obtained by the trial subject was accurate, the newly-diagnosed case felt comfortable receiving his result from a partner in a nonmedical setting, and the case altered his behavior to reduce ongoing HIV transmission despite the absence of post-test counseling. On the other hand, although the case received the correct preliminary result, he was unaware that he needed confirmatory testing and delayed seeking care. The absence of post-test counseling also meant that the trial subject, whose identity remains unknown to us, was unaware of the availability of and recommendation for post-exposure prophylaxis.

Although in this case the partners had already engaged in unprotected sex, partners may choose to test together to inform sexual decision-making. Many MSM serosort (i.e. choose to have sex only with partners of concordant HIV status, to use condoms only with discordant partners, or to have HIV-negative partners be the insertive partner in discordant partnerships) based on what partners disclose directly or indirectly [13]. If MSM trust the results of home-use tests and are therefore more likely to serosort, the ability for sex partners to test at home could increase unprotected sex and allow for transmission of other STIs. OraQuick’s relatively long window period [14] could also increase false-negative results during primary infection and lead to unprotected sex when individuals are most infectious. However, the results of home-use tests may be more accurate than self-reported HIV status and may therefore decrease unprotected sex between serodiscordant partners [15]. Another concern is that the availability of home-use tests may result in coerced testing or intimate partner violence, particularly if partnerships are newly identified as serodiscordant.

This case report relied on an interview with the case and therefore may be limited by recall error and social desirability bias.

## Conclusions

This case demonstrates that home-use tests can be used to successfully identify HIV infection and that they will likely be used by MSM to test sex partners. This case also highlights concerns about the absence of post-test counseling and the potential for delayed linkage to care among those who receive reactive home-use tests. The U.S. National HIV/AIDS Strategy emphasizes immediate linkage to care [16] because delaying care can adversely impact the HIV-infected individual’s health [17] and ongoing transmission [2]. Additional research must be done to determine under what circumstances home-use tests can be used to increase awareness of HIV status, how they impact linkage to care among persons newly diagnosed with HIV, and whether home-use tests can be safely used to increase the accuracy of serosorting.

## Consent

Written informed was obtained from the case for publication of this Case report. A copy of the written consent is available for review by the Series Editor of this journal.

## Abbreviations

MSM: Men who have Sex with Men; STIs: Sexually Transmitted Infections; FDA: Food and Drug Administration.

## Competing interests

The author(s) declare that they have no competing interests.

## Authors’ contributions

DK conceived of the study, conducted the interview with the case, and drafted the manuscript. JS and MG participated in the design of the study and helped draft the manuscript. All authors read and approved the final manuscript.
